# Who’s at Risk and Why? Multilevel Factors of Later-Life Financial Fraud in China

**DOI:** 10.1093/geroni/igaf122.3145

**Published:** 2025-12-31

**Authors:** Ting Hu, Flavia Andrade, Yu-Chih Chen

**Affiliations:** University of Illinois--Urbana-Champaign, Champaign, Illinois, United States; University of Illinois--Urbana-Champaign, Urbana, Illinois, United States; National Taiwan University, Taipei, Taipei, Taiwan (Republic of China)

## Abstract

Older adults are particularly vulnerable to financial fraud. However, research on risk factors of later-life fraud has focused more on individual attributes without considering contextual factors. Additionally, these studies fail to examine diverse types and amounts of financial loss when measuring financial fraud. We address these gaps using a comprehensive framework to explore the multilevel risk factors of fraud in later life. Using 8,151 participants aged 57+ from the 2015-2018 China Health and Retirement Longitudinal Study, we employed mixed-effects models to examine how factors regarding suitable targets (e.g., previous fraud experience, socioeconomic status, cognition, etc.), capable guardians (e.g., marital status, social participation, etc.), and motivated offenders (e.g., locality, available financial institutions, GDP, etc.) were associated with exposure, types of victimization, and amount of financial loss. Findings showed that prior fraud experience was linked to all three measures of financial fraud. Better episodic memory and greater social activity were associated with higher fraud exposure, while better mental intactness and greater social activity were linked to lower financial loss. Additionally, contextual vulnerabilities—such as urban areas and more developed regions (e.g., more financial institutions and tertiary sectors or Eastern/Central areas)—were associated with increased fraud exposure or financial loss. Our findings highlight the need to target older adults living in urban and Eastern/Central areas, particularly those with prior fraud experiences, better cognitive well-being, higher socioeconomic status, and active social lives. Program and policy efforts related to anti-scam hotlines, educational workshops, and psychological support should be established to combat later-life financial fraud.

